# Left-handed surgical instruments – a guide for cardiac surgeons

**DOI:** 10.1186/s13019-016-0497-9

**Published:** 2016-08-19

**Authors:** Clare Burdett, Maureen Theakston, Joel Dunning, Andrew Goodwin, Simon William Henry Kendall

**Affiliations:** Department of Cardiothoracic Surgery, James Cook University Hospital, Marton Road, Middlesbrough, TS4 3BW UK

**Keywords:** Left-handed, Lefthanded, Left handed, Surgical instruments, Cardiothoracic, Surgery, Surgical training

## Abstract

For ease of use and to aid precision, left-handed instruments are invaluable to the left-handed surgeon. Although they exist, they are not available in many surgical centres. As a result, most operating theatre staff (including many left-handers) have little knowledge of their value or even application. With specific reference to cardiac surgery, this article addresses the ways in which they differ, why they are needed and what is required - with tips on use.

**Electronic supplementary material:**

The online version of this article (doi:10.1186/s13019-016-0497-9) contains supplementary material, which is available to authorized users.

## Background

Many surgical instruments do not express laterality (for example forceps), but some important ones do. Where this is the case, the best instruments for a left-handed surgeon are those designed for left-handed use. In hospitals, the ‘generic’ equipment provided is actually tailored to the needs of the right-hander. Accounting for less than 10 % of the workforce, most left-handers are pragmatic and adapt to using right-handed equipment where they can. However, compromise is not always possible; surgery requires fine motor skills and some aspects of an operation demand a very high degree of precision and ‘dexterity’. In such circumstances, it is necessary to utilise the most efficient and comfortable tools for the job. Having access to left-handed equipment early in a surgeon’s career greatly facilitates their training. Left-handed equipment does exist, but is not routinely stocked in the operating theatre [1]. As a result, knowledge and experience of left-handed instruments is limited to a few centres.

## How they differ

Subtle differences in their construction make an instrument right or left-handed. These differences have a significant impact on how the instrument works when placed in the surgeon’s hand. For a right-handed piece of equipment the natural (often subconscious) force that a right-hander applies to the instrument cannot be replicated by the left-hander without deliberate thought. An unnatural position needs to be assumed which is less intuitive being inefficient for the muscle groups involved, and will preclude deftness in execution [2].

In broad terms surgical instruments can be split into 4 categories: cutting, clamping, grasping and retracting. The instruments with laterality either have ratchets (some clamping, grasping and retracting instruments) or blades (cutting). The more precise the task for which they are used, the greater need there is for tailoring them to the surgeon’s handedness.

### Needle holders

Most needle holders have a ratchet. This is a ‘mechanism’ designed to lock an instrument when it is closed. To ensure it remains secure, only force in one direction will unlock it. Toothed serrations are present on both shanks of the needle holder, and mirror each other so that they lock.

**Fig. 1:**
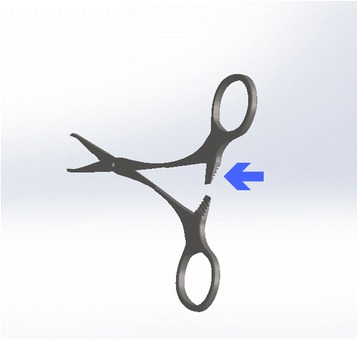
Right-hander using a right-handed needle holder

**Fig. 2:**
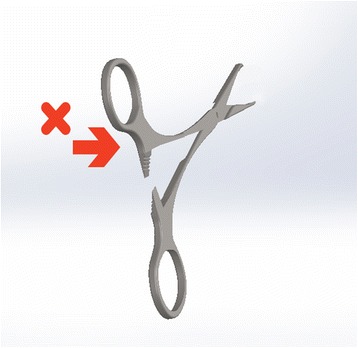
Left-hander using a right-handed needle holder

**Fig. 3:**
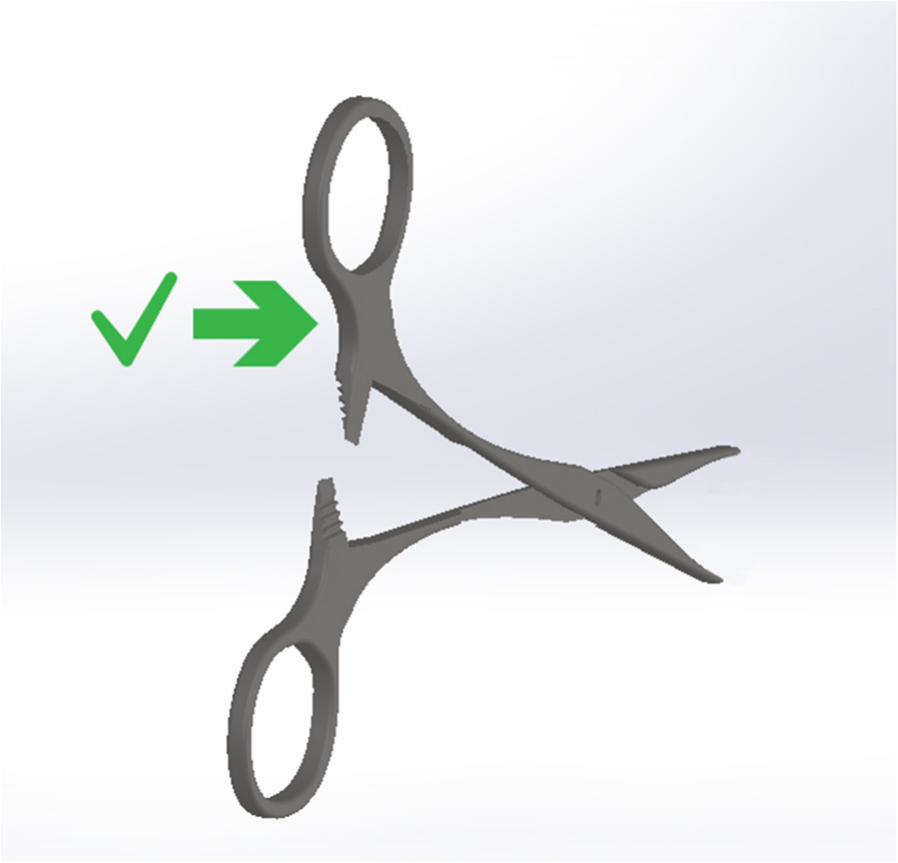
Left-hander using a left-handed needle holder

The toothed serrations of the upper shanks of a right-handed instrument form the left side of the ratchet. A simple push of the upper handle by the surgeon’s right thumb will displace the teeth further to the left away from their right-sided counterparts (Fig. 1). Placed in the left-hand, the same pushing action by the thumb is now from the left and pushes the upper handle towards the right, where its teeth are already locked in place with their opposite counterparts (Fig. 2). To overcome this, the left-hander must instead ‘pull’ the upper shank to the left, away from the lower shank to free the teeth. This is a less natural and controllable movement. With a left-handed instrument, the upper limb has its ratchet mechanism to the right, allowing the left-handers thumb to push it away from its toothed counterpart with ease (Fig. 3).

Many other instruments have ratchets (for instance, mosquitos and cross-clamps) and these pose the same problem. However, having the correct ratchet is more important for needle holders because the work is delicate and the action of locking and unlocking is repeated many times whilst stitching. For equipment where opening the ratchet is less frequent and the task is not so refined, left-handers can usually work with right-handed equipment. This saves cost and permits easy transfer of instruments between left and right-handers at the operating table. However, the modified left-handed action will never look as slick.

### Scissors

Right-handed scissors have the right blade on top whichever way up you hold them. This has two consequences: −

Held in the right-hand, the surgeon has a clear view of the line they are cutting. Held in the left-hand, the upper blade obscures this view owing to the position of the scissors relative to the operator’s midline. Inevitably the left-hander is disadvantaged at maintaining accuracy. To overcome this a left-hander would need to move their left hand across their body and hold the scissors to the right of the midline, which would be less comfortable and inefficient for the muscle groups involved. Alternatively, they can angle the top blade away from themselves but as a result, they are cutting at a slant. Left-handed scissors have the left blade on top to give the left-hander a perfect view of the cutting line.

**Fig. 4:**
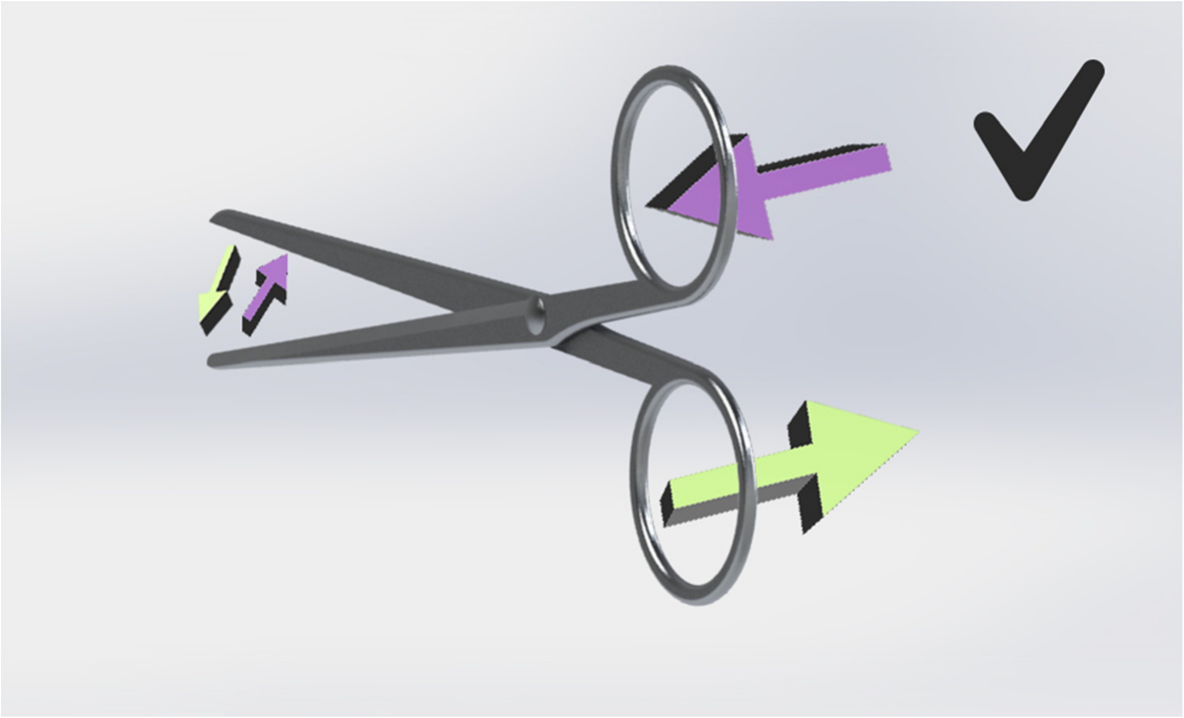
Right-hander using right-handed scissors

**Fig. 5:**
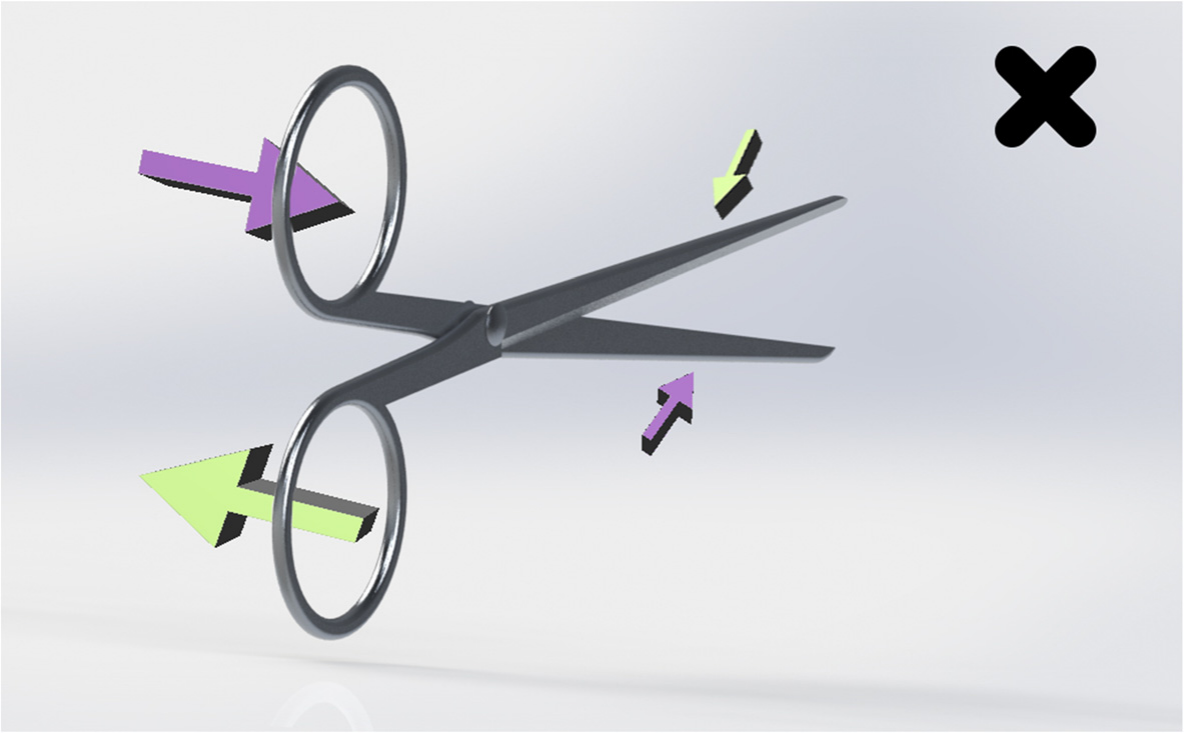
Left-hander using right-handed scissors

**Fig. 6:**
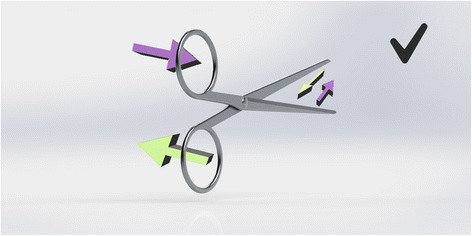
Left-hander using left-handed scissors

Scissors cross at the hinge. Therefore, the top handle controls the bottom blade (and vice versa). However, their action is not purely vertical. Instead they are designed to have lateral give at the hinge, so that the direction of force above the hinge results in the opposite direction below it. The natural, subconscious action of the hand is to gently push with the thumb and pull with the fingers as you close a pair of scissors. This has been utilised in the design of right-handed scissors to ensure the blades make contact as they pass each other. In the right hand, the right blade is uppermost. It is controlled by the finger, which slips through the lower handle and pulls it to the right, causing the blade to move toward the left. The thumb approaching from the right pushes the top handle to the left, moving the lower (left-sided) blade to the right. So, the right blade moves left and left blade moves right bringing the two blades close together to make a clean cut (Fig. 4). If the left hand is used, the finger still operates the upper blade, which is still the right-sided scissor. Now the pulling action on the handle is towards the left so the blade moves towards the right. The thumb, now approaching the handle from the left pushes it towards the right, which makes the lower (left) blade move leftwards. The result is, the right blade moves right and the left blade moves left (Fig. 5). The resultant gap causes the material being cut to fold between the blades [3]. A cut may still be achieved through the shearing force applied, but it will not be as accurate or clean and cannot be guaranteed. This effect is poorer still with blunt scissors or a loose hinge, which exaggerates the gap. Left-handers compensate by placing the thumb far into the ring of the upper handle so that they can pull (rather than push) it, whilst jamming their finger against the inside wall of the lower ring to push rather than pull. This is an unnatural movement requiring force. Smaller hands are less effective at achieving this. Cutting thicker material can require a great deal of force and some degree of folding between the blades is likely. Left-handed scissors have the blades reversed to eliminate this problem (see Fig. 6).

## Specifics for cardiac surgery

The required repertoire of operative techniques varies between specialties with emphasis on different skills. In cardiac surgery, the following are of particular note for the left-hander:

### Needle control

Cardiac surgeons operate within the confined space of the chest cavity. Often their hands are located outside the cavity but the surgical instrument they hold extends downwards in to the chest. Needle control and positioning must be maintained at a distance. Distance magnifies false movements. Pulling on a right-handed ratchet to force it open is not a smooth action and can lessen control of the needle and even displace it.

### Palming

Palming is a technique used to increase the degree a needle holder can be manipulated by allowing it to rotate further around its axis. When a thumb is placed in the finger ring the axis of rotation becomes off centre, causing lateral stress on the tissue as the needle passes through. With palming, the axis is improved allowing the needle to rotate cleanly through the tissue. This prevents the needle cutting through and minimises the size of the needle hole.

The technique of palming evokes strong opinions, with some centres in favour and others against. One certainty is that in centres promoting its use, to not do so is considered tantamount to failure as a surgeon. This can be an early stumbling block for many left-handed trainees. Palming requires the needle holder to be held in the palm rather than placing fingers through the finger rings. For the right-hander, the ratchet can be opened by a simple push from the thenar eminence. In the left hand, a pushing motion does not open the ratchet. Instead it needs to be pulled, an action that cannot be achieved using the thenar eminence (Additional file 1). A few left-handed surgeons manage to get part of their thumb above the top finger ring to pull it, but this does not give optimal control and can only be achieved by those with large hands. For palming the left-hander needs a left-handed needle holder (Additional file 2).

### Suturing with fine needle holders

**Fig. 7:**
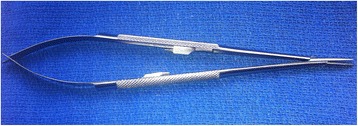
Fine needle holder

The fine needle holders used for coronary anastomoses (micro-vascular needle holders e.g. *Castroviejo* or *Jacobson*) do not pose the same difficulties (see Fig. 7). Their ratchets are universal as they can be locked both ways and require little force to overcome. If preferred, non-ratcheted versions are available. Some centres use a larger version of this style of needle holder (with jaws big enough to accommodate larger needles) for a substantial part of the operation, including set-up (e.g. cannulation purse strings) and valve replacement. When these are used, the issue of palming does not arise.

### Scissors

In the assistant role, cutting is mainly limited to sutures which can often be achieved using right-handed scissors. As scissors pass regularly between different operators at the table, this compromise keeps life simple for both you and the scrub nurse. Scissors that are blunt or loose at the hinge are less forgiving and may present a problem during a case. All assistants need to be able to use both hands, so learning how to use scissors right and left is helpful for simple tasks.

As first operator, the situation is more complicated. A pair of right-handed scissors that are in good condition may be acceptable for fine tissues that separate easily, although left-handed scissors would be preferable. For tougher or thicker tissues the result from using right-handed scissors can be poor, with tissue folding between the blades. If using scissors to cut skin (for instance when harvesting leg vein) the result with right-handed scissors will be inferior and you should insist on left-handed scissors for cosmeses.

## Manufacture

Many makers of surgical instruments will produce left-handed equipment if asked, even if it is not stated in their brochure or website. Producing left-handed equipment takes longer. The standard manufacturing process is set up to produce right-handed equipment as they form the majority of sales. For left-handed equipment, stamped blanks cannot be passed through the normal process. Instead the ratchet or blade has to be individually cut and welded into the correct position. As a result, left-handed instruments cost approximately 50 % more to produce, an up-lift reflected in their price.

## Access and logistics

Provision of left-handed equipment costs extra money. It is not expensive when compared to other hospital expenditures, but it is often viewed as inessential, particularly when left-handers pass through the department intermittently. As a result it is usually unavailable to new trainees. Obtaining it can also be difficult. As there are no clear guidelines on who should meet the cost – the training body, the hospital or the surgeon. It is therefore currently purchased on a case-by-case basis.

Consistency in using left or right-handed equipment is important because of the muscle memory that develops. Switching between right and left needle holders is counter-productive. As a result, continuous access to left-handed instruments must be assured. In recent years, concerns over infection control have prevented hospitals accepting equipment already used elsewhere. This restriction particularly impacts on the junior surgeon who is likely to move between hospitals for their training quite frequently. Either, all teaching hospitals should maintain left-handed sets, or barriers to their movement need to be addressed. Senior (consultant) surgeons move less often, but logistical issues can still arise for them too. For instance, their opportunities to help out in an emergency or take up sessions in other hospitals (public or private) can be restricted.

## The essential kit

Below are examples of the basic instrument sets that a left-hander should have in theatre. The standard set would still be opened to provide the generic equipment.

### Cardiac set

- 2 × 7-inch left-handed *Mayo* needle holder
- 2 × 7-inch left-handed *Debakey* needle holder
- 2 × 9-inch left-handed long *Debakey* needle holder
- 1 × 7-inch left-handed *Berry* sternal wire needle holder.

### Optional-

- 1 × 7-inch left-handed *Metz* scissors
- 1 × 7-inch left-handed *Mayo* scissors.

### Leg vein set

- 1 × 7-inch left-handed *Mayo* scissors
- 1 × 7-inch left-handed *Mayo* needle holder.

### Optional -

- 1 × 7-inch left-handed *Metz* scissors.

Some instruments require 2 in each set, so that the scrub nurse can load the second whilst the first is in use. The number of sets required will depend on the number of cases that a surgeon is likely to do per day. Ideally, an additional set should be available for emergencies or in case one of the sets (or equipment within it) has to be withdrawn from use at short notice. For most centres this equates to 3 or 4 sets per left-handed surgeon or trainee.

## Tips for use

### For the scrub nurse

#### Preparation

Often, left-handed equipment is in short supply. If so, check with the surgeon what equipment they need open initially and what can be kept nearby in the operating theatre but remain unopened. Also consider whether instruments should be fast-tracked for cleaning at the end of the case.

#### Opening left-handed needle holders

The right-handed scrub nurse will need to place their thumb through the top handle of the needle holder and pull it away from the midline (and slightly up) to open it.

### Loading needles

This often causes confusion but is actually straightforward. A left-hander’s forehand is the same as a right-handers backhand, and vice versa. If you are used to working with right-handers and their terminology, just load for the opposite shot to what has been requested or you are predicting is next.

### Scissors

If the left-hander is going to be using right-handed scissors make sure they are sharp and tight. Remember, the trainee is not being awkward if they hand them back and request another pair.

### For the trainee surgeon

#### Act with confidence

Don’t apologise for being left-handed. Many left-handed trainees get used to being told that they, rather than the instruments, are at fault. Use the information herein to inform others as to why certain pieces of equipment don’t work and make a case for purchasing left-handed equipment. Also, remember that sometimes equipment really is just faulty!

#### Preparation

Make sure you turn up early and speak to the scrub nurse before the case. If you have not worked together previously you may need to show them how to open needle holders and load the needle. Even when you work regularly with a scrub nurse, discuss what you need and ensure they have left-handed equipment out for you. If you are not sure how much of the case you will be doing, keep potentially required items unopened but close at hand in the theatre. This ensures that you are always prepared for an ad hoc training opportunity.

#### Using equipment

Be consistent when using left or right-handed equipment. For instance, you might use left-handed needle holders but persist with right-handed scissors for assisting. Whatever you decide, stick to your chosen routine. Switching left for right throughout the case will confuse the scrub nurse causing hesitation and precluding them from anticipating what you require next. It will also take longer for you to learn a new skill.

#### Familiarising yourself with left-handed equipment

Be aware initially, that you may transfer adaptive movements you have developed for right-handed equipment to your new left-handed instruments. For instance, if you are used to putting your thumb far into the handle of a pair of scissors and pulling, you may initially transfer this to a left-handed pair of scissors. This will cause the blades to move apart and you will be just as frustrated with the left-handed equipment as you previously were with the right. Persevere – the movements are more natural, so you will pick them up quickly. Once you get used to left-handed equipment, you won’t want to go back.

## Conclusions

For the left-handed surgeon, left-handed instruments are a pleasure to use. Subtle differences in design make a big difference to performance. Previously thought out tasks become subconscious, each move is more efficient and positioning is comfortable.

## Additional files

Additional file 1: Left-hander attempting to open a right-handed needle holder. (13.5 MB) Additional file 2: Left-hander palming with a left-handed needle holder. (13.2 MB)
